# Overexpression of Interleukin-33 in Recombinant Rabies Virus Enhances Innate and Humoral Immune Responses through Activation of Dendritic Cell-Germinal Center Reactions

**DOI:** 10.3390/vaccines10010034

**Published:** 2021-12-28

**Authors:** Zhizhong Mi, Ling Zhao, Ming Sun, Ting Gao, Yong Wang, Baokun Sui, Yingying Li

**Affiliations:** 1 College of Basic Medicine, Dali University, Dali 671000, China; mizhizhong1994@163.com (Z.M.); elagwf@163.com (M.S.); gt742620585@163.com (T.G.); ayxwy76@163.com (Y.W.); 2 State Key Laboratory of Agricultural Microbiology, Huazhong Agricultural University, Wuhan 430070, China; lingzhao@mail.hzau.edu.cn (L.Z.); suibaokun@mail.hzau.edu.cn (B.S.)

**Keywords:** rabies vaccine, interleukin-33, dendritic cells, innate immune response, germinal center reaction, humoral immune response

## Abstract

Rabies is a zoonotic infectious disease caused by rabies virus (RABV), and its mortality rate is as high as 100%. Globally, an average of 60,000 people die from rabies each year. The most effective method to prevent and limit rabies is vaccination, but it is currently expensive and inefficient, consisting of a 3-dose series of injections and requiring to be immunized annually. Therefore, it is urgent to develop a single dose of long-acting rabies vaccine. In this study, recombinant rabies virus (rRABV) overexpressing interleukin-33 (IL-33) was constructed and designated as rLBNSE-IL33, and its effect was evaluated in a mouse model. The results showed that rLBNSE-IL33 could enhance the quick production of RABV-induced immune antibodies as early as three days post immunization (dpi) through the activation of dendritic cells (DCs), a component of the innate immune system. Furthermore, rLBNSE-IL33 induced high-level virus-neutralizing antibodies (VNA) production that persisted for 8 weeks by regulating the T cell-dependent germinal center (GC) reaction, thus resulting in better protection against rabies. Our data suggest the IL-33 is a novel adjuvant that could be used to enhance innate and humoral immune responses by activating the DC-GC reaction, and thus, rLBNSE-IL33 could be developed as a safe and effective vaccine for animals.

## 1. Introduction

Rabies is an acute zoonotic infectious disease caused by Rabies virus (RABV), and is a negative-stranded RNA virus belonging to the *Lyssavirus* genus of the *Rhabdoviridae* family [1]. RABV is transmitted through animal bites, and almost all human rabies deaths are due to dog bites [2,3]. Due to the lack of effective treatment, the mortality rate of human rabies is close to 100% [4]. Therefore, preventive measures against rabies are of great importance. At present, the main means of prevention and control of rabies is vaccination. The main vaccination used in humans and animals is safe inactivated rabies vaccines. However, the price is expensive and the immune effect is not desirable. In addition, multiple immunizations are required over an extended period of time (14 days) to obtain good protective effect [5]. Therefore, it is urgent to develop a new affordable and more effective rabies vaccine for rabies control. Live-attenuated rRABVs can efficiently provide antibodies to protect the body against RABV after a single dose, and thus have the potential to be developed as an ideal vaccine.

IL-33 is a member of the IL-1 family, and it can effectively drive the production of cofactor T helper 2 cells (Th2). IL-33 is one of the ligands of ST2 (suppression of tumorigenicity 2). In the inactive state, IL-33 in its prosomatic form (full length 270 amino acid residues) is chromatin-related cytokine [6]. Recent evidence has suggested that IL-33 can proliferate and differentiate dendritic cells (DCs) [7,8], macrophages [9] and T cells [10]. IL-33 can activate the signal transducer and activator of transcription 4 (STAT4), up-regulate the expression of B-cell lymphoma 6 (Bcl6), and promote the differentiation of CD4^+^CXCR5^+^ (cysteine-X-cysteine chemokine receptor type 5) Tfh (Follicular helper T) cells [11]. Tfh cells are essential for the formation of germinal centers (GCs) which trigger Tfh cells to maintain and regulate the differentiation of GC B cells to form plasma cells (PCs) and memory B cells (Bmem) [12,13,14]. This indicates that IL-33 may activate innate immunity by regulating the maturation of DCs, further inducing GC reactions, eventually improving humoral immunity after RABV immunization, suggesting that IL-33 has the potential to be applied as a molecular adjuvant.

In this study, we constructed a rRABV expressing murine IL-33 (rLBNSE-IL33) and evaluated its innate and humoral immunity effect in a mouse model. Our results indicate that rLBNSE-IL33 can significantly induce early and high levels of VNA production by increasing DCs, Tfh cells, GC B cells, and PCs. By activating the DC-GC reaction, rLBNSE-IL33 can produce more antibodies to improve the protection against rabies.

## 2. Materials and Methods

### 2.1. Cells, Viruses, Antibodies, and Animals

The BSR cells were cloned from BHK-21 cells, maintained in Dulbecco’s modified Eagle’s medium (DMEM) (Gibco, Grand Island, NY, USA) containing 10% fetal bovine serum (FBS) (Gibco, Grand Island, NY, USA) and antibiotics (100 units/mL Penicillin and 100 µg/mL Streptomycin) (Beyotime, Wuhan, China). Mouse neuroblastoma (NA) cells were maintained in Roswell Park Memorial Institute (RPMI)-1640 medium (Gibco, Grand Island, NY, USA) containing 10% FBS and antibiotics (100 units/mL Penicillin and 100 µg/mL Streptomycin) (Beyotime, Wuhan, China). The rRABV strain LBNSE was derived from SAD-B19 by removing the pseudogene and adding *BsiW*I and *Nhe*I sites between G and L genes, with mutation at 194 and 333 position of amino acid of G protein. Rabies challenge virus strain 24 (CVS-24) was reproduced from 5-day-old suckling mouse brains. The following antibodies were purchased from Biolegend (San Diego, CA, USA), of which FITC-CD11c (clone N418), APC-CD80 (clone 16-10A1), PE-CD86 (clone GL-1) were used for flow cytometry analysis of dendritic cells; FITC-CD4 (clone GK1.5), APC-CXCR5 (clone L138D7), PE-PD-1 (clone RMP1-30) for flow cytometry analysis of Tfh cells; PE-Cy7-B220 (clone RA3-6B2), Alexa Fluor647-GL7 (clone GL7), and PE-CD95 (APO-1/Fas) (clone 15A7) for flow cytometry analysis of GC B cells; PE-Cy7-B220 and APC-CD138 (clone 281-2) for flow cytometry analysis of plasma cells. The following antibodies were used for immunofluorescence assay of lymph nodes (LNs). Alexa Fluor647-CD45R/B220 (clone RA3-6B2), Alexa Fluor488-IgG (clone Poly4053), and Streptavidin-Alexa Fluor594 were purchased from Biolegend (San Diego, CA, USA), and GL7 (clone GL7) was purchased from eBioscience (San Diego, CA, USA). These antibodies were used for the detection of RABV-specific antibody isotypes, including horseradish peroxidase (HRP)-conjugated goat anti-mouse immunoglobulin G (IgG), IgG1, IgG2a, IgG2b, IgG3, and IgM (Boster, Wuhan, China). All animals were purchased from the Hunan SJA Laboratory Animal Company (Hunan, China). Our animal experiments followed the protocols of the Scientific Ethics Committee of Dali University (with protocol number of DLDXLL2020-0508).

### 2.2. Construction and Rescue of rRABV Expressing Murine IL-33

The murine IL-33 cDNA was amplified from RNA extract of mouse brain infected by RABV using RT-PCR, and then inserted between G and L genes of pLBNSE to replace the deleted non-coding pseudogene, resulting in the production of pLBNSE-IL33. The primers used for IL-33 PCR amplification included forward primer 5′-GTA TAC GTA CGA TGA GAC CTA GAA TGA AGT ATT C-3′ and reverse primer 5′-CTA GCT AGC TTA GAT TTT CGA GAG CTT AAA C-3′ with *BsiW*I and *Nhe*I sites underlined. The clone of pLBNSE-IL33 and four helper plasmids containing the N, P, G, and L genes of LBNSE were transfected into BSR cells by using SuperFect (Qiagen, Valencia, CA, USA). The rescued rLBNSE-IL33 was detected using FITC-conjugated RABV N protein-specific antibodies.

### 2.3. Virus Titration

A direct fluorescent antibody test was performed to determine virus titers in BSR cells. BSR cells were incubated with a ten-fold serially diluted rRABVs in 96-well plates at 37 °C for 48 h. Subsequently, the incubated cells were washed with 300 μL phosphate-buffered saline (PBS) three times, then fixed with 80% ice-cold acetone and stained with FITC-conjugated RABV N protein-specific antibodies for 1 h. Antigen-positive fluorescent spots were counted as focus-forming units per milliliter (FFU/mL) under Olympus IX51 fluorescence microscope (Olympus, Tokyo, Japan). Virus dilution at each concentration was added into four wells.

### 2.4. Cell Viability Assay

The BSR cells were incubated, respectively, with LBNSE (positive control), rLBNSE-IL33 (experiment group), and DMEM (as mock) at a multiplicity of infection (MOI) = 1 in 96-well plates at 37 °C for 48 h. After incubation, the infected cell supernatant was collected. Cell activity was detected with the cell proliferation assay kits (Promega, Madison, WI, USA) following the manufacturer’s instructions. The absorbance under excitation light at 490 nm was detected using a Tecan infinite 200 microplate tester (Mannedorf, Switzerland). 

### 2.5. IL-33 Concentration Determination through ELISA

The BSR cells were incubated, respectively, with LBNSE and rLBNSE-IL33 at a MOI of 0.001, 0.01, 0.1, and 1 in 96-well plates until the convergence of cells reached 80%–90%. After 24 h infection, the supernatants were collected to detect the IL-33 expression with commercially available mouse IL-33 ELISA kits (RayBiotech, Atlanta, GA, USA) following the manufacturer’s instructions. The absorbance under excitation light at 450 nm was tested with a Tecan infinite 200 microplate tester (Tecan, Mannedorf, Switzerland). 

### 2.6. Mouse Immunization and Challenge Test

Six-week-old female ICR mice injected, respectively, with 100 µL of LBNSE (10^6^ FFU), rLBNSE-IL-33 (10^6^ FFU), and DMEM (as mock) via an intramuscular (i.m.) route were divided into three groups. All the infected mice were challenged with 30 µL of 50× lethal dose 50 (LD50) CVS-24 via an intracerebrally (i.c.) route at 21 dpi and observed daily for three weeks. 

### 2.7. Pathogenicity Determination

The six-week-old female ICR mice were injected, respectively, with 30 µL of LBNSE (10^7^ FFU), rLBNSE-IL-33 (10^7^ FFU), and DMEM (as mock) via i.c. route. Five-day-old ICR mice were injected with 10 µL of LBNSE (100 FFU), rLBNSE-IL-33 (100 FFU), and DMEM (as mock) via i.c. route, respectively. All the mice body weight and survival rates were examined twice daily for three weeks. 

### 2.8. Flow Cytometry Assay

Flow cytometry was used to detect the cells in LN and bone marrow (BM) of immunized mice. LN cells or BM cells were made into suspension through a 40 µm nylon filter, then washed with PBS, and centrifugated. After centrifugalization, the obtained cells were added with 0.2% bovine serum albumin (BSA) and fluorescence-conjugated antibodies and incubated for 30 min at 4 °C in the dark. The samples were washed twice with PBS containing 0.2% BSA and resuspended. The 300 μL cell suspensions (10^5^ cells/sample) were subjected to a flow cytometry assay with a BD FACSCalibur (BD, Piscataway, NJ, USA).

### 2.9. Immunofluorescence Assay

LNs were cut into 35 µm section by a Themo Cryotome FSE freezing microtome (Themo, Cheshire, UK). After being washed with PBS three times, the sections were incubated with primary antibodies for 10 h at 4 °C in the dark, then washed with PBS three times, and incubated with the second antibodies for 2 h at room temperature. After being washed with PBS three times, the sections were placed on glass slides and observed under an Olympus IX51 fluorescence microscope (Olympus, Tokyo, Japan).

### 2.10. Virus-Neutralizing Antibody (VNA) Test

Blood samples of the mice were collected at different time points post immunization, and the level of VNA was determined by fluorescent antibody virus neutralization (FAVN) tests. The prepared 50 μL three-fold diluted experiment samples and standard serum samples (National Institute for Biological Standards and Control, Herts, UK) were added into 96-well plates. Each well was added with 50 μL CVS-11 suspension containing 100 FFU, and the samples were incubated at 37 °C, 5% CO_2_ for 1 h, added with 50 μL BSR cells (5 × 10^5^ cells/mL) and incubated at 34 °C, 5% CO_2_ for three days. Then each well was added with 100 μL ice cold acetone (pre-cooled at −20 °C) and incubated at room temperature for about 30 min and air-dried. Afterwards, the samples were stained with FITC-conjugated anti-RABV N antibodies for 45 min at 37 °C, and then washed three times with PBS. The resultant samples were observed under an Olympus IX51 fluorescence microscope (Olympus, Tokyo, Japan). We compared the fluorescence values of our prepared serum samples with those of standard samples to obtain VAN titers, which were expressed as international units per milliliter (IU/mL).

### 2.11. RABV-Specific Antibody Isotype Test 

ELISA was used to detect and analyze the RABV-specific antibody isotypes. The 96-well ELISA plates were coated with 100 μL Na_2_CO_3_ buffer (5 mM, pH 9.6) containing RABV G protein (0.5 µg/well) (Boster, Wuhan, China) for 12 h at 4 °C. The plates then were washed three times by PBS-Tween and blocked with 5% skimmed milk buffer for 2 h at 37 °C. Serum samples collected from the immunized mice were diluted at a ratio 1:30 of serum to PBS-Tween, then the dilution was added into each well and incubated for 2 h at 37 °C. After incubation, the plates were washed three times with PBS-Tween and then incubated with diluted HRP-conjugated goat anti-mouse IgG (1:1000), IgG1 (1:1500), IgG2a (1:1500), IgG2b (1:2000), IgG3(1:1500), or IgM (1:2000) (Boster, Wuhan, China) for 45 min at 37 °C, respectively. Then the plates were washed three times with PBS-Tween, added with 100 µL tetra-methyl-benzidine (TMB) substrate (Boster, Wuhan, China), and incubated in the dark for 30 min at 37 °C. Then the reaction was stopped by adding 2M H_2_SO_4._ The absorbance at 450 nm was measured with a Tecan infinite 200 microplate tester (Tecan, Mannedorf, Switzerland).

### 2.12. Statistical Analysis

All data were analyzed by GraphPad Prism 8.0 software (GraphPad Software, lnc., San Diego, CA). Kaplan–Meier survival curves were analyzed by the log rank test for the percent survival experiments. Significant differences between the groups in two independent experiments were determined by an unpaired two-tailed *t*-test (parametric test), and were indicated at three levels: *, *p* < 0.05; **, *p* < 0.01; and ***, *p* < 0.001.

## 3. Results

### 3.1. Construction and Expression of rLBNSE-IL33

**Figure 1:**
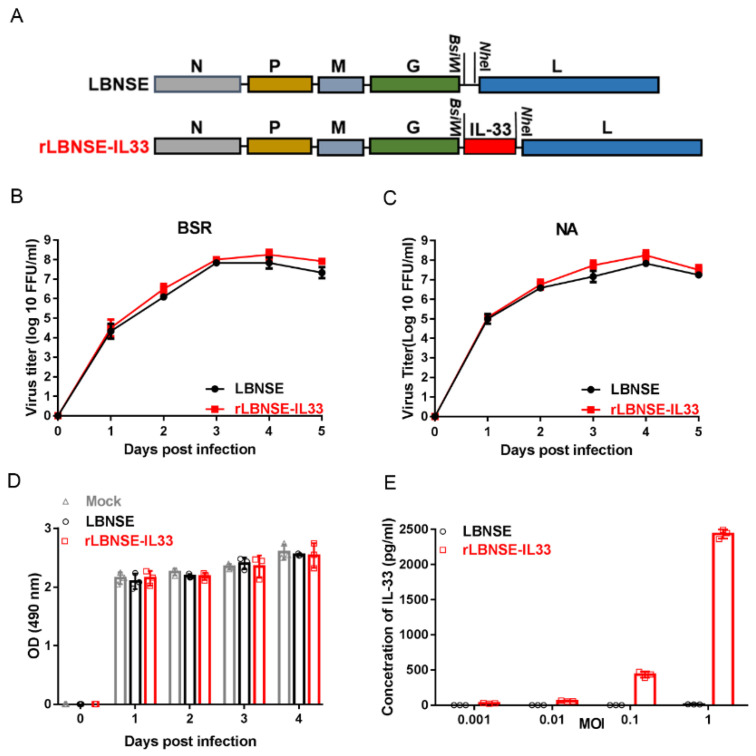
Construction and characterization of rLBNSE-IL33 in vitro: (**A**) schematic representation for LBNSE and rLBNSE-IL33 construction. The parent vector pLBNSE was derived from SAD-B19 strain with long non-coding region between G and L genes deleted, and at the deletion region, restriction enzyme sites of BsiWI and NheI were introduced. RABV nucleoprotein, phosphoprotein, matrix, glycoprotein, and polymerase genes were represented by N, P, M, G, and L, respectively. Murine IL-33 was acquired and inserted in between G and L gene of RABV genome. Supernatants of LBNSE and rLBNSE-IL33 infected cells at the MOI of 0.01 were collected to determine the titers of LBNSE and rLBNSE-33 at 1, 2, 3, 4 and 5 dpi; (**B**,**C**) virus multiple-step growth curve in BSR cells and NA cells respectively; (**D**) viability of BSR cells at 1, 2, 3 and 4 dpi. (**E**) IL-33 expression level in the infected cells by ELISA. Error bars indicated standard deviation (SD, *n* = 3).

In order to determine the role of IL-33 in the immunogenicity of RABV, we cloned the murine IL-33 gene, inserted it between G and L genes of LBNSE vector, and obtained rLBNSE-IL33 (Figure 1A). RT-PCR and sequencing results confirmed that the inserted IL-33 gene was stable in at least ten consecutive passages in BSR cells (data not shown). The BSR cells (Figure 1B) and NA cells (Figure 1C) were infected, respectively, with LBNSE and rLBNSE-IL33 at a MOI of 0.01 to draw the multiple-step growth curves. The curves of rLBNSE-IL33 in BSR cells and NA cells were similar to those of the parent strain LBNSE, indicating that the insertion of IL-33 had no significant effect on the viral replication in vitro. The cell viability assays of BSR cells revealed that the viability of rLBNSE-IL33-infected cells was similar to that of LBNSE-infected cells (Figure 1D). ELISA results indicated that in LBNSE or rLBNSE-IL33 infected BSR cells at different MOIs, the production of IL-33 was dose-dependent (Figure 1E).

### 3.2. rRABVs Pathogenicity in Mice

**Figure 2:**
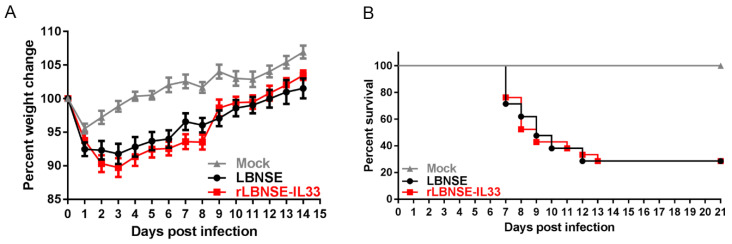
rRABVs pathogenicity in mice: (**A**) weight of six-week-old ICR female mice (*n* = 10) i.c. infected with LBNSE (10^7^ FFU), rLBNSE-IL-33 (10^7^ FFU), or DMEM (as mock); (**B**) survival rates of five-day-old suckling ICR mice (*n* = 21) i.c infected with LBNSE (100 FFU), rLBNSE-IL-33 (100 FFU) or DMEM (as mock). The error bars indicated standard error (SE).

Since the safety of rRABVs was important, we evaluated the possible adverse effects of rLBNSE-IL33 on animals. Three groups of six-week-old female ICR mice (*n* = 10) were i.c. infected with LBNSE (10^7^ FFU), rLBNSE-IL-33 (10^7^ FFU), or DMEM (as mock). The body weights were measured for 14 days consecutively. No mice were manifested with any rabies-related clinical symptoms. The weight curve of mice infected with rLBNSE-IL-33 exhibited little difference from the weight curve of mice infected with LBNSE (Figure 2A). Three groups of five-day-old suckling ICR mice (*n* = 21) were i.c. injected with 100 FFU of rRABVs to examine the effect of rLBNSE-IL-33 on immuno-compromised mice model pathogenicity and their survival rates were recorded every day. The results showed that no significant difference in survival rates was observed between the rLBNSE-IL-33-injected mice and the LBNSE-injected mice (Figure 2B). Taken together, the above results indicated that the overexpression of IL-33 of rRAVB in vivo had no side effects.

### 3.3. Overexpression of IL-33 in rRABV Promotes Activation of Dendritic Cells (DCs)

**Figure 3:**
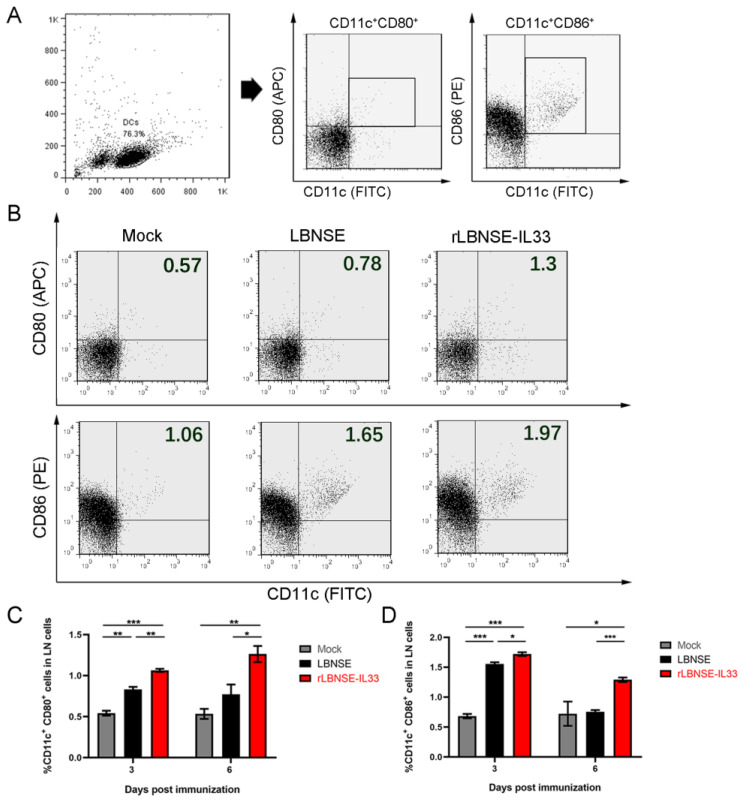
Overexpression of IL-33 in rRABV promotes the activation of DCs: (**A**,**B**) cell populations and gating strategies of DCs based on flow cytometry analysis. The inguinal LNs from mice i.m. immunized with LBNSE (10^6^ FFU), rLBNSE-IL33 (10^6^ FFU), or DMEM (as mock) were collected and made into 10^5^ single-cell suspensions at 3 and 6 dpi, and were labeled by antibodies for analyzing DCs (CD11c^+^CD86^+^, CD11c^+^CD80^+^); (**C**,**D**) percentage of activated DCs per 10^5^ draining LN cells in three immunized groups at 3 dpi (**C**) and 6 dpi (**D**). The standard errors (SE, *n* = 3) was illustrated by error bars, and the data were analyzed by unpaired two-tailed *t*-test. Significant differences between groups were indicated at three levels: *, *p* < 0.05; **, *p* < 0.01; and ***, *p* < 0.001.

IL-33 can promote DC maturation and recruit DCs to draining LNs [7,8]. In order to investigate whether IL-33 overexpression can promote the activation of DCs in vivo, six-week-old BALB/c mice were i.m. injected, respectively, with LBNSE (10^6^ FFU), rLBNSE-IL33 (10^6^ FFU), and DMEM (as mock), and the inguinal LNs were collected at 3 and 6 dpi. Flow cytometry analysis of 10^5^ individual inguinal LN cells was performed to determine the activation of DCs (CD11c^+^CD80^+^, CD11c^+^CD86^+^) (Figure 3A). The results indicated that the percentage of DCs in the mice vaccinated with rLBNSE-IL33 was significantly higher than that vaccinated with LBNSE at 3 and 6 dpi (Figure 3C,D). Overall, these results indicated that the immunization with IL-33-overexpressing rRABV enhanced the activation of DCs.

### 3.4. Overexpression of IL-33 in rRABV Facilitates Growth and Development of Draining LNs

**Figure 4:**
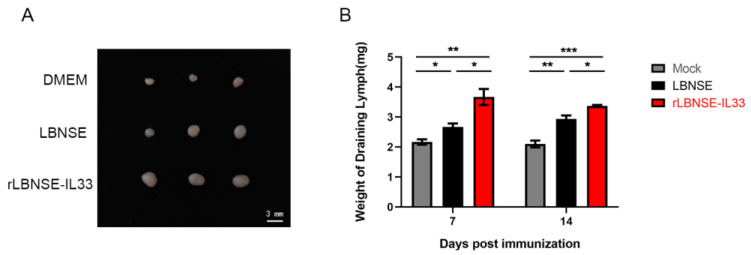
Overexpression of IL-33 in rRABV facilitated the growth and development of draining LNs. The inguinal LNs were collected from mice i.m. injected respectively with LBNSE (10^6^ FFU), rLBNSE-IL33 (10^6^ FFU), and DMEM at 7 and 14 dpi. The sizes (**A**) and weights (**B**) of these LNs were measured. The standard error (SE, *n* = 3) was illustrated by error bars, and the data were analyzed by unpaired two-tailed *t*-test. Significant differences between groups were indicated at three levels: *, *p* < 0.05; **, *p* < 0.01; and ***, *p* < 0.001.

Since the secondary lymphoid organs are essential for high-affinity antibody production in the immune system [15], we further investigated the effect of overexpression of IL-33 on the growth and development of draining LNs in this study. BALB/c mice received LBNSE (10^6^ FFU), rLBNSE-IL33 (10^6^ FFU) or DMEM (as mock) via i.m. route, and the inguinal LNs were collected and weighed at 7 and 14 dpi. As shown in Figure 4A, the LNs from mice immunized with rLBNSE-IL33 were obviously bigger than those from mice immunized with LBNSE. The LNs’ weight from rLBNSE-IL33-vaccinated mice was significantly higher than that from LBNSE-vaccinated mice at 7 and 14 dpi (Figure 4B). These results suggested that the immunization with IL-33 overexpressing RABV could promote the growth and development of draining LNs.

### 3.5. Overexpression of IL-33 in rRABV Induces Generation of Tfh Cells

**Figure 5:**
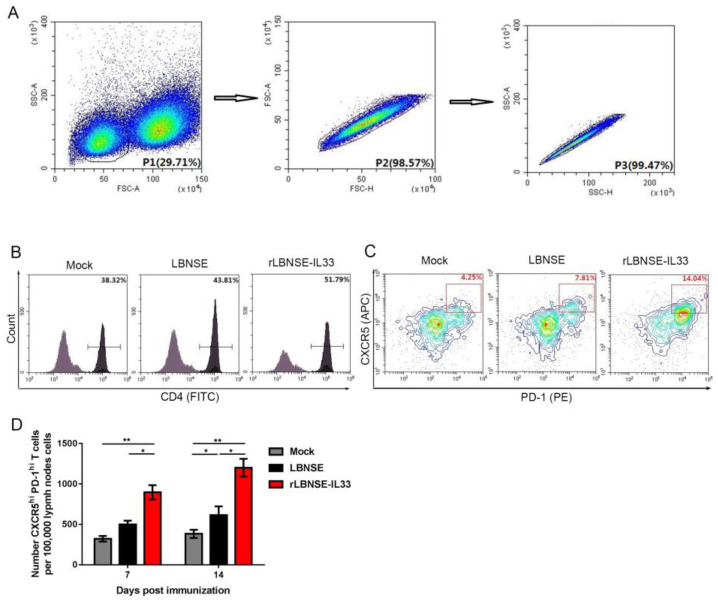
Overexpression of IL-33 by rRABV induced the generation of Tfh cells. Inguinal LNs from six-week-old BALB/c mice i.m. injected with LBNSE (10^6^ FFU), rLBNSE-IL33 (10^6^ FFU), or DMEM were collected and made into 10^5^ single-cell suspensions at 7 and 14 dpi, and then the suspensions were stained with antibodies for flow cytometry analysis: (**A**) target Cells were visualized with dot plots to show their forward-scatter (FSC) and side-scatter (SSC) signals respectively representing size and granularity of the cells; (**B**,**C**) gating strategies and contour plots respectively representing the CD4^+^T cells in lymphocytes (**B**) and Tfh cells in CD4^+^T cells (**C**); (**D**) total number of Tfh cells per 10^5^ draining LN cells at 7 and 14 dpi. The standard error (SE, *n* = 3) was illustrated by error bars, and the data were analyzed by unpaired two-tailed *t*-test. Significant differences between groups were indicated at the levels: *, *p* < 0.05 and **, *p* < 0.01.

IL-33 has been reported to induce Tfh cells to enhance humoral immunity against hepatitis B virus infection [11]. In order to investigate the effect of IL-33 overexpression in rRABV on the generation of Tfh cells, six-week-old BALB/c mice (*n* = 3) were i.m. injected with rRABVs (10^6^ FFU) or DMEM (as mock). Flow cytometry analysis of 10^5^ individual inguinal LN cells were performed to determine the number of lymphocytes at 7 and 14 dpi (Figure 5A). The gating strategies were used to detect CD4^+^T cells (Figure 5B) and Tfh cells (CD4^+^CXCR5^hi^PD-1^hi^) (Figure 5C) in flow cytometry analysis, as previously described [16,17]. Our results showed that there were more Tfh cells in LNs from mice immunized with rLBNSE-IL33 than those immunized with LBNSE at 7 and 14 dpi (Figure 5D). Taken together, these data suggested that the immunization with IL-33-overexpressing rRABV could enhance the generation of Tfh cells. 

### 3.6. Overexpression of IL-33 in rRABV Enhances Expansion of GC B Cells 

**Figure 6:**
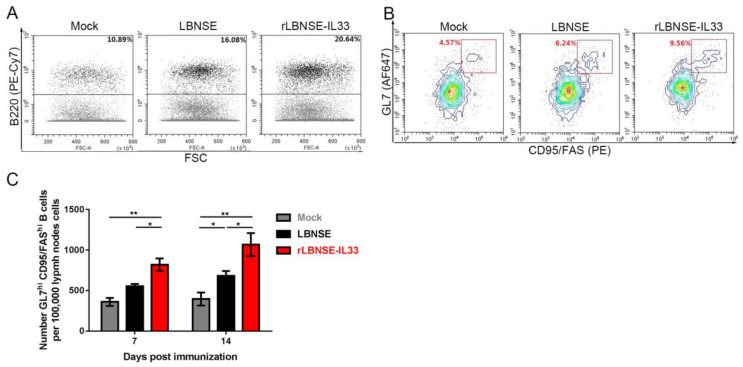
Overexpression of IL-33 in rRABV enhances the expansion of GC B Cells. The suspensions of inguinal LNs from mice immunized with rRABVs were obtained and stained by antibodies for flow cytometery analysis of GC B cells (B220^+^GL7^hi^CD95/Fas^hi^) at 7 and 14 dpi: (**A**,**B**) number of B220^+^ B cells in lymphocytes (A) and number of GC B cells in the B220^+^ B cells (**B**) based on flow cytometric strategies; (**C**) Total number of GC B cells per 10^5^ draining LN cells at 7 and 14 dpi. The standard error (SE, *n* = 3) was illustrated by error bars, and the data were analyzed by unpaired two-tailed *t*-test. Significant differences between groups were indicated at the levels: *, *p* < 0.05 and **, *p* < 0.01.

Tfh cells have been reported to contribute to the generation of GC B cells [13]. In this study, the effect of IL33 overexpression in rRABV on GC B cell production was further investigated. Inguinal LNs were collected from immunized BALB/c mice at 7 and 14 dpi and made into single-cell suspensions. The representative gating strategies were used to detect B220^+^B cells (Figure 6A) and GC B cells (B220^+^GL7^hi^CD95/Fas^hi^) (Figure 6B) in lymphocytes. The number of GC B cells collected from rLBNSE-IL33-immunized mice was significantly larger than that from LBNSE-immunized mice, indicating that IL-33 played an important role in the expansion of GC B cells.

### 3.7. Overexpression of IL-33 in rRABV Promotes Formation of GCs

**Figure 7:**
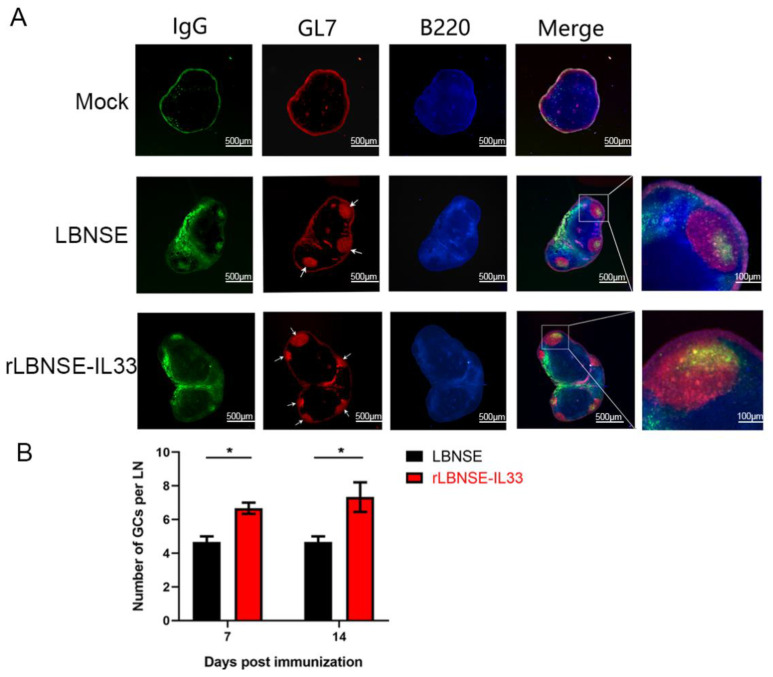
Overexpression of IL-33 in rRABV promotes the formation of GCs. Six-week-old female BALB/c mice were vaccinated via i.m. route with 10^6^ FFU rRABVs or DMEM. The draining LNs were collected and stained (IgG, green; GL7, red; and B220, blue) for GC number detection: (**A**) representative results of immunofluorescence represented GCs in LNs; (**B**) number of GCs per LN was counted at 7 and 14 dpi. The standard error (SE, *n* = 3) was illustrated by error bars, and the data were analyzed by unpaired two-tailed *t*-test. Significant differences between groups were indicated at the levels: *, *p* < 0.05.

The interaction between Tfh cells and B cells produces high-affinity antibodies in the GCs [18]. In this study, the effect of IL-33 overexpression in rRABV on GCs formation was further evaluated. The inguinal LNs were collected from female BALB/c mice immunized with LBNSE (10^6^ FFU), rLBNSE-IL33 (10^6^ FFU), or DMEM (as mock) via i.m. route at 7 and 14 dpi. The GC formation in LNs was detected by immunofluorescence assay. Representative staining results of GC formation at 7 dpi in mice immunized with different rRABVs were shown in Figure 7A. As shown in Figure 7B, the number of GCs in LNs from mice immunized with rLBNSE-IL33 was significantly larger than that from mice immunized with LBNSE at 7 and 14 dpi. The above results demonstrated that IL-33 overexpression could enhance the formation of GCs post immunization.

### 3.8. Overexpression of IL-33 in rRABV Increases Number of Plasma Cells (PCs)

**Figure 8:**
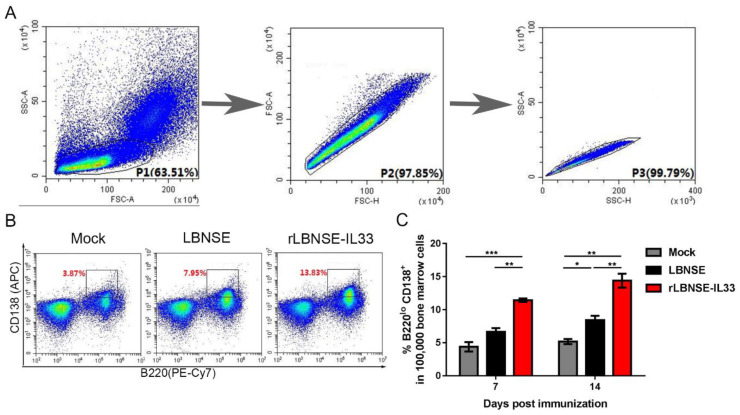
Overexpression of IL-33 in rRABV increases the PC number. Six-week-old female BALB/c mice were infected with LBNSE (10^6^ FFU), rLBNSE-IL-33 (10^6^ FFU), or DMEM (as mock) and their BM cells were collected at 7 and 14 dpi: (**A**) gating strategies and contour plots of lymphocytes in BM cells; (**B**) flow cytometric analysis of PCs (B220^lo^CD138^+^) in the lymphocytes of BM cells; (**C**) percentage of PCs per 10^5^ BM cells at 7 and 14 dpi. The standard error (SE, *n* = 3) was illustrated by error bars, and the data were analyzed by unpaired two-tailed *t*-test. Significant differences between groups were indicated at three levels: *, *p* < 0.05; **, *p* < 0.01; and ***, *p* < 0.001.

PCs are differentiated from GC B cells, and they could produce antibodies against viral infection [19]. In order to investigate the effect of rLBNSE-IL33 on PCs generation, BM cells were obtained from immunized female BALB/c mice injected with LBNSE (10^6^ FFU), rLBNSE-IL33 (10^6^ FFU), or DMEM (as mock), and then PCs in BM cells were detected by flow cytometry. The gating strategies for the detection of PCs (B220^lo^CD138^+^) were shown in Figure 8A,B. The results showed that the percentage of PCs in the rLBNSE-IL33-immunized mice was significantly higher than that in the LBNSE-immunized mice at 7 and 14 dpi, which suggested that overexpression of IL-33 in rRABV can increase the number of PCs (Figure 8C).

### 3.9. Overexpression of IL-33 in rRABV Improves Antibody Responses and Protection against Rabies

**Figure 9:**
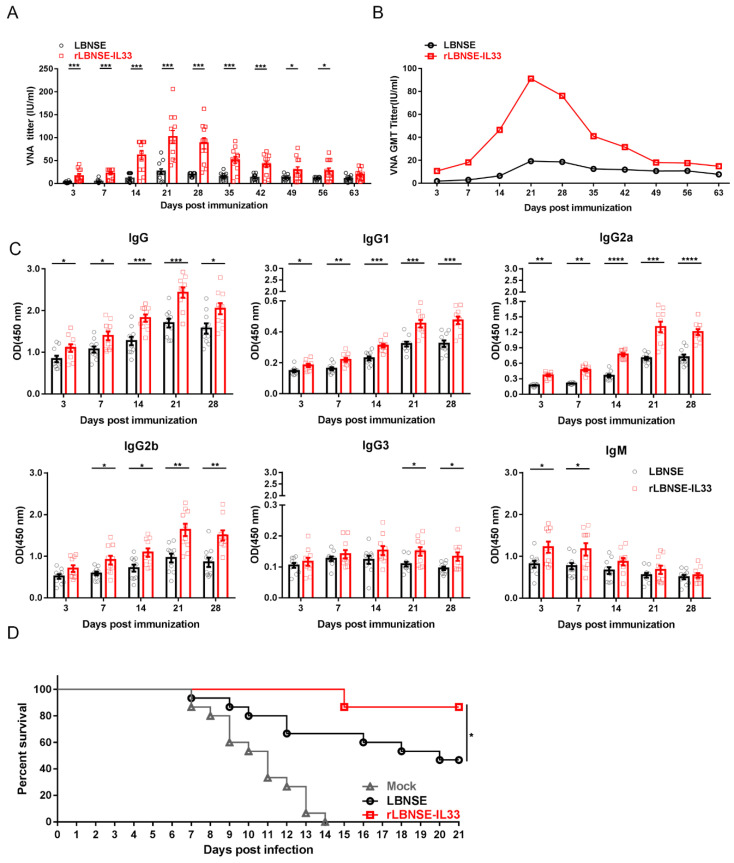
Overexpression of IL-33 in rRABV improved antibody responses and protection against rabies. Six-week-old female ICR mice were immunized with LBNSE (10^6^ FFU), rLBNSE-IL33 (10^6^ FFU), or DMEM (as mock) via i.m. route. Serum samples of vaccinated mice (*n* = 10) were harvested and analysed by FAVN test at each indicated time points for nine weeks: (**A**) VNA level; (**B**) GMT; (**C**) absorbance at 450 nm of RABV-specific IgG, IgG1, IgG2a, IgG2b, IgG3, and IgG M antibodies based on ELISA. At 21 dpi, the immunized ICR mice (*n* = 15) were challenged with 50 × LD_50_ of CVS-24 via i.c. route, and monitored daily for three weeks; (**D**) survival rate after the challenge. The standard error (SE, *n* = 3) was illustrated by error bars, and the data were analyzed by unpaired two-tailed *t*-test. Significant differences between groups were indicated at three levels: *, *p* < 0.05; **, *p* < 0.01; ***, *p* < 0.001 and ****, *p* < 0.0001.

To determine whether the significantly increased PCs in BM cells promoted anti-RABV antibody production after rLBNSE-IL33 vaccination, six-week-old female ICR mice (*n* = 10) were immunized with LBNSE (10^6^ FFU), rLBNSE-IL33 (10^6^ FFU) or DMEM (as mock) via i.m. route. Serum samples of mice were collected at each indicated time points post immunization to measure the levels of VNA by FAVN tests. The results indicated that VNA levels in the mice immunized with rLBNSE-IL33 were obviously higher than those in the mice immunized with LBNSE from 3 to 56 dpi (Figure 9A), and the geometric mean titer (GMT) corresponding to VNA levels showed the similar trend (Figure 9B). The rLBNSE-IL33-immunized mice exhibited an early dramatic increase in VNA level (10.74 IU/mL) at 3 dpi. The highest VNA level was 91.19 IU/mL in the rLBNSE-IL33-immunized group, and that was 19.33 IU/mL in the LBNSE-immunized group at 21 dpi. After 63 dpi, no significant difference in the VNA level was detected between these two groups. ELISA was used to detect the RABV-specific antibody isotypes in the serum samples. As expected, immunization with rLBNSE-IL33 significantly induced the high levels of IgG, IgG1, and IgG2a at all the time points from 3 to 28 dpi (Figure 9C). 

The protection test was performed to evaluate the effect of rLBNSE-IL33 immunization on protection against rabies. All the immunized ICR mice (*n* = 15) were challenged with 50 × 50% LD_50_ of CVS-24 via i.c. route and monitored daily for three weeks from 21 dpi. As shown in Figure 9D, all the mock-immunized mice succumbed to rabies within two weeks, while 86.67% of rLBNSE-IL33-immunized mice and 46.67% of LBNSE-immunized mice survived.

Taken together, overexpression of IL-33 in rRABV could significantly induce early antibody response and high levels of antibody production and effective protect mice against rabies.

## 4. Discussion

Our studies found that overexpression of IL-33 in rRABV promotes serial DC-GC responses, inducing innate and humoral immune responses after RABV immunization. Results indicated that rLBNSE-IL33 could significantly enhance VNA production as early as 3 dpi by activating DC maturation in innate immunity and by maintaining high levels of VNAs for eight weeks by regulating the T cell-dependent GC response, thus resulting in a better protection against rabies. As reported by previous studies, several attempts have been made to explore that overexpression of cytokines in RABV as a recombine vaccine to enhance the immunogenicity. The rRABVs overexpressing a cytokine such as macrophage inflammatory protein-1 alpha (MIP-1α) [20], fms-like tyrosine kinase 3 ligand (Flt3L) [21], IL-7 [22], and IL-21 [23] have been reported to enhance immune responses by mainly activating DC or GC response, but compared with our results, their antibody response is relatively short-term and slow.

Safety is the primary criterion for evaluating vaccines. In our study, the multiple-step growth curves and recorded body-weight changes and survival rates proved the rRABVs’ safety. The pathogenicity investigation results indicated a slight decrease in body weight in mice infected with rLBNSE-IL33 at the first 9 dpi, but such a decrease was not statistically significant, which might be due to the transient inflammatory response induced by IL-33 [24]. This inflammatory response could be easily overcome by the immune system, thus the mice weight quickly started to increase. Additionally, no significant difference in the survival rate was observed between mice infected with rLBNSE-IL33 and those infected with LBNSE. The parent strain LBNSE has been reported to generate from the SAD-B19 mutation at the amino acid sites of G protein 194 and 333, resulting in the nonpathogenicity of LBNSE via i.m. route and oral administration [25]. In summary, these results suggest that rLBNSE-IL33 as a rabies vaccine is safe.

As the main antigen-presenting cells in the body, DCs can effectively connect the innate immune recognition of viruses and acquired immune response to viruses in a CD8^+^ T cell-dependent manner [26]. The interaction between mature DCs and T cells or B cells is the basis for inducing the acquired immune response [27]. Previous studies have shown that rRABV expressing the stimulating factor of DCs can activate host innate immunity by recruiting and activating DCs, rapidly inducing a high level of early virus neutralizing antibody (VNA) production and improving its immunogenicity [28,29]. However, the production of antibody is slow and transient in existing studies. In our study, the rLBNSE-IL33-immunized mice exhibited a dramatic increase in VNA level as early as 3 dpi and high level VNA was maintained for 8 weeks. The high level VNA production and maintenance as well as the activation of DCs by rLBNSE-IL33 may be due to the synergic action of ST2 (suppression of tumorigenicity 2), MyD88 (myeloid differentiation factor88) and STAT1 (signal transducer and activator of transcription to induce costimulatory molecule expression of DCs [7]. Based on these findings, it can be concluded that the recruiting and activation of DCs by IL-33-overexpressing rRABVs can induce innate immune response to promote early antibody production after immunization.

After DCs present antigen to T cells, CD4^+^T cells can differentiate into Tfh cells, and Tfh cells promote the formation of GCs, thus developing and differentiating into PCs and memory B cells [30]. Considering the important role of Tfh cells in inducing immune response, immunologists have proposed that Tfh cells can be a potential target for optimizing vaccine design [12]. Correspondingly, in our study, we found that mice infected with rLBNSE-IL33 could produce more Tfh cells, which might be attributed to the activation of STAT4, which can promote the differentiation of CD4^+^CXCR5^+^ Tfh cell by activating Bcl6 expression induced by IL-33 [11]. The IL-33 receptor ST2 and the chemokine receptor CXCR5 are markers for the differentiation and maturation of Tfh cells, and IL-33/ST2 pathway represents a major target of these markers and Bcl6 [31]. Besides, a previous study has found one type of cell population from mice exposed to ovalbumin (OVA) with IL-33, and the cell could highly express Bcl6 and Tcf7 (encoding TCF-1), a recently reported upstream transcription factor of Tfh cells [32].

Our study found that overexpression of IL-33 in rRABV increased the number of GC B Cells, which might be due to the enhanced generation of Tfh cells, which provided stimulus signals to GCs [33,34]. Tfh cells can mediate high affinity B cells’ cloning in GC and regulate the size and quality of GC during maturation [12]. Tfh cells interact with B cells through CD40/CD40L (cluster of differentiation) [11]. One previous study has reported that the activation of ST2 by IL-33 could activate CD40L signaling in a cancer mice model [35]. IL-33 plays an important role in inducing the development of GCs. Chronic exposure to IL-33 has been reported to elicit dramatic elevation in B-cell activating factor (BAFF) levels, thus increasing the numbers of B cells and Tfh cells and promoting GC formation in humoral immunity [36]. Interestingly, the rRABV-based vaccine expressing BAFF induced similar anti-RABV antibody responses [37]. In addition, one study of an adeno-associated virus in a mice model has found that transient exposure to IL-33 could increase the number of GCs in combination with the induction of cytokines such as MCP-1, MIP-1α, and MIP-1β [38]. However, the related mechanism still needs to be further explored. Lymphoid tissue inducer (LTi) cells are a subgroup of innate lymphoid cells (ILC), and LTi cells can activate stromal organizer cells to produce chemokines and adhesion molecules during the development of LN [39]. Considering that IL-33 can activate group 2 ILC (ILC2s) via induction of the tryptophan hydroxylase 1 (Tph1) [40,41], we speculated that IL-33 might promote the growth and development of draining LNs by activating LTi cells. However, the corresponding mechanism remains to be further investigated.

IL-33 has been reported to be expressed in lymphocytes and PCs, suggesting that IL-33/ST2 might participate in immune responses by regulating miR-524-5p [42]. PCs are differentiated from GC B cells, and they can further produce antibodies over time [43]. The high-level VNA production and maintenance and large number of RABV G-specific antibody isotypes in our study might be due to the increased number of PCs. RABV G is the primary target of VNA, and RABV G-specific antibodies, especially IgG, IgG2a, and IgG2b, are associated with prolonged protective antibody responses post RABV immunization; IgM is associated with the high level of early antibody response [44]. Taken together, overexpression of IL-33 in rRABV could induce a relatively long-term immune response.

## 5. Conclusions

In conclusion, overexpression of IL-33 in rLBNSE-IL33 could enhance innate and humoral immune responses to induce a quick and long-lasting antibody response and a more effective protection against rabies by recruiting and activating DCs, generating more Tfh cells, GC B cells, PCs, and enhancing the formation and growth of GC. This study confirmed the safety and high efficiency of rLBNSE-IL33, thus rLBNSE-IL33 can be developed as an effective and uninjurious vaccine for animals.

## Data Availability

The research data can be acquired on request from the correspondence author.
